# Sex Differences in Asthma Pathogenesis

**DOI:** 10.1111/imr.70100

**Published:** 2026-01-08

**Authors:** Kaitlin E. McKernan, Emely Henriquez Pilier, Dawn C. Newcomb

**Affiliations:** ^1^ Department of Pathology, Microbiology, and Immunology Center for Vanderbilt Immunobiology Nashville Tennessee USA; ^2^ Department of Medicine Vanderbilt University Medical Center Nashville Tennessee USA

**Keywords:** asthma, inflammation, sex differences, sex hormones

## Abstract

There is a sex difference in asthma prevalence and asthma‐related morbidity that changes from childhood into different reproductive stages of life. The impact of sex hormone signaling on asthma pathogenesis has been partially elucidated using large cohort human studies, human cells, and animal models. Androgens decreased airway inflammation by reducing type 2 inflammation and eosinophil infiltration as well as reducing neutrophil‐induced airway inflammation in animal models. Estrogen signaling through ER‐α increased IL‐33 production, an alarmin produced by airway epithelial cells that increases type 2 inflammation and increased Th17 cell‐mediated neutrophilic inflammation. Additional studies are needed to determine what happens to asthma control and asthma‐induced inflammation in women during pregnancy and menopause as well as how sex differences in immune cell development and airway inflammation affect asthma incidence and onset during childhood. Collectively, understanding that sex differences in asthma risk and control exist throughout life is important to personalize therapies for males and females with asthma.

Sex differences in inflammation occur at the disease level down to the cellular level. As children, boys have increased incidence of inflammation, infections, and asthma compared to girls [1, 2]. As adults, women have increased prevalence and incidence of lupus, multiple sclerosis, and asthma; men have increased rates of non‐reproductive cancer malignancies [1, 2]. These sex differences in the prevalence and risks of inflammatory disease exist throughout life, yet asthma risks shift from being more pronounced in boys during childhood to becoming a higher prevalence in women as adults. Women also have increased numbers of asthma exacerbations compared to men [2]. Cohort clustering analysis using a large asthma cohort analysis study—the Unbiased Biomarkers for the Prediction of Respiratory Disease Outcomes (UBIOPRED) cohort—showed that women with asthma had increased exacerbation rates and more frequent visits to urgent cares or emergency departments compared to men with asthma [3]. In this review, we will discuss the sex differences in asthma and how sex hormones and sex chromosomes drive mechanisms that lead to differences in asthma prevalence, severity, and symptoms in females and males in different stages of life.

## 1 Pathogenesis of Asthma

**FIGURE 1:**
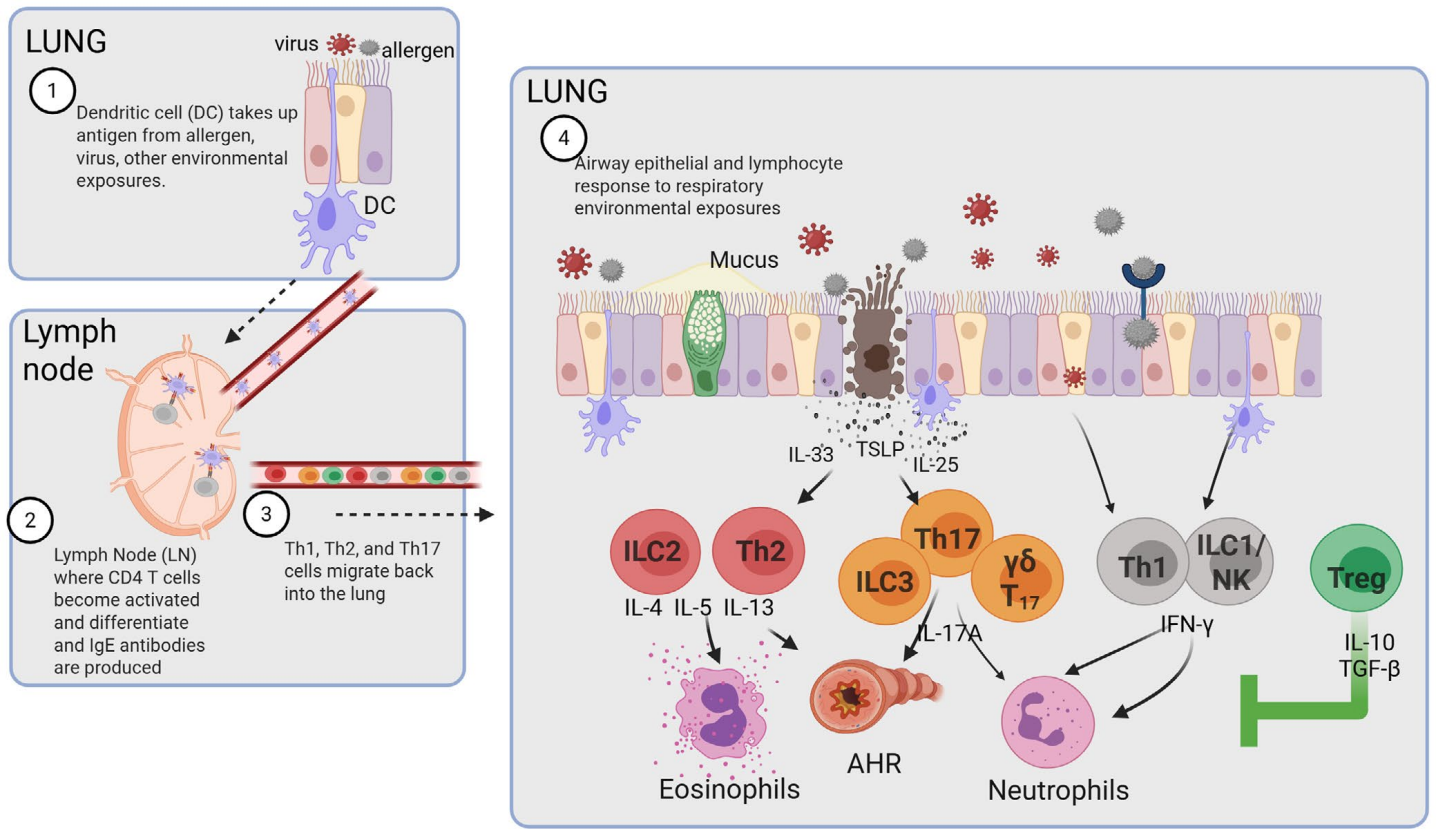
Schematic of lung inflammation in asthma pathogenesis.

To understand how sex hormones and sex chromosomes drive inflammation, one must first understand the mechanisms that drive airway inflammation, mucus production, and airway hyperresponsiveness—the hallmarks of asthma. Inflammation in asthma is caused by many different mechanisms in response to environmental exposures, including aeroallergens, pollutants, and respiratory viruses. Additionally, asthma is not one disease of chronic inflammation, rather an umbrella disease with many different phenotypes and mechanisms driving inflammation [4, 5, 6, 7]. As shown in Figure 1, eosinophil infiltration into the airway is the result of type 2‐mediated airway inflammation. Quickly upon exposure to an aeroallergen or other environmental exposure, alarmin cytokines—IL‐33, TSLP, and IL‐25—are released to potentiate and stimulate downstream type 2 immune responses. Type 2 immune cells, including type 2 conventional dendritic cells, take up antigen, migrate to the lung draining lymph nodes, and activate CD4+ T cells to become Th2 differentiated cells. Th2 cells then migrate back to lung to produce IL‐4, IL‐5, and IL‐13, leading to infiltration of eosinophils, increased mucus production from airway epithelial cells (AECs), and increased AHR. Back in the lung draining lymph nodes, T follicular 13 cells educate B cells to produce IgE antibodies [8, 9, 10] further leading to allergic inflammatory response. In the lung, group 2 innate lymphoid cells (ILC2) also become stimulated and produce high levels of IL‐5 and IL‐13 with lower levels of IL‐4. All this leads to increased type 2 lung inflammation.

**FIGURE 2:**
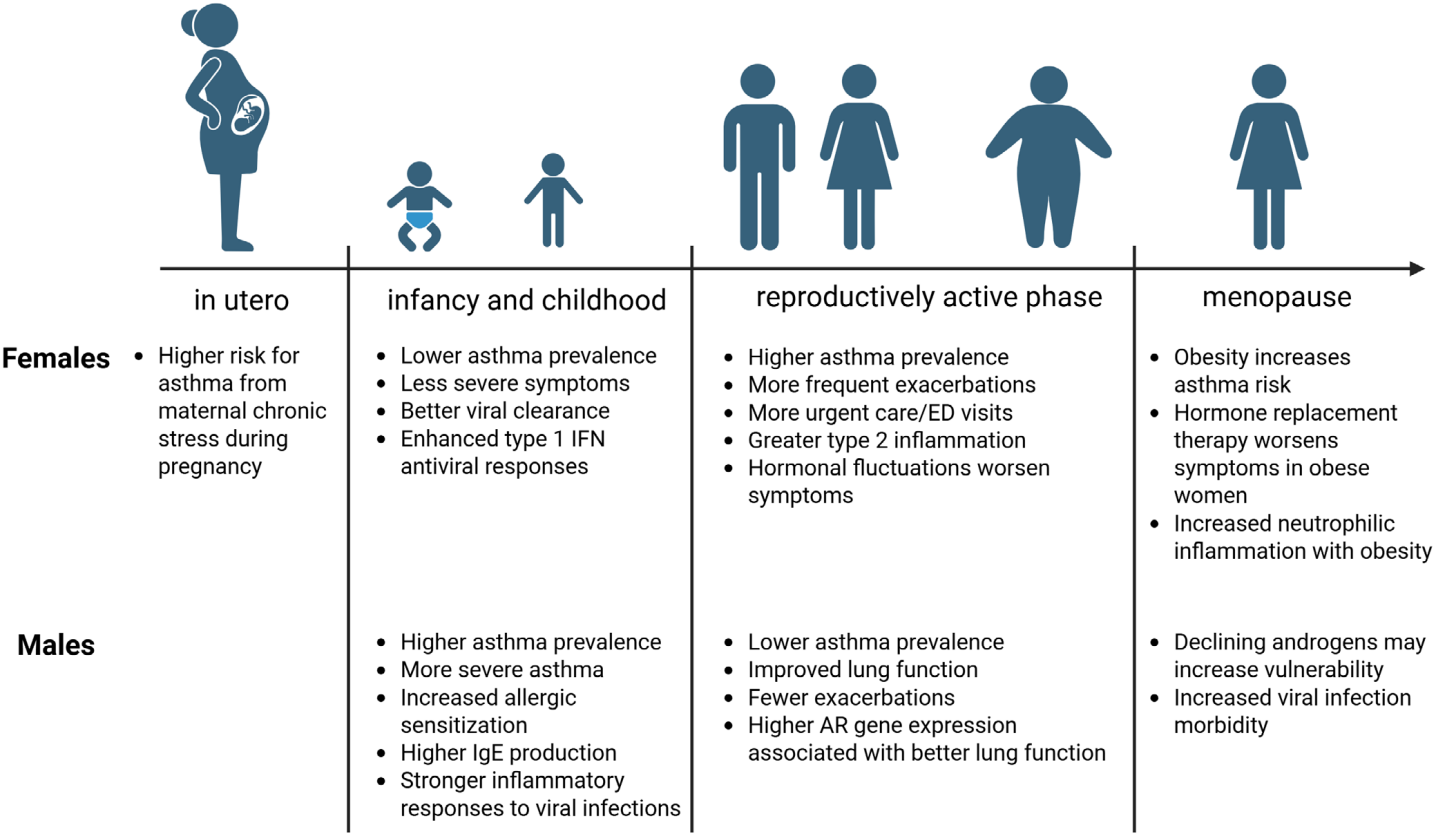
Summary of findings at different life stages for asthma risk, prevalence, or asthma pathogenesis.

In some patients, non‐type 2 inflammatory responses are also observed with increased neutrophils [11, 12, 13, 14]. Neutrophilic infiltration is driven by increased IFNγ and/or IL‐17A production, and IFNγ and IL‐17A were increased in bronchoalveolar lavage (BAL) fluid and in CD4 and CD8 T cells of patients with severe asthma compared to controls or milder asthma phenotypes [15, 16, 17, 18, 19, 20, 21]. IL‐17A is produced by Th17 cells, γδ T cells, and ILC3s, and IFNγ is produced by CD8+ T cells, Th1 cells, γδ T cells, ILC1s, and NK cells [22, 23, 24]. Increases in non‐type 2 inflammation are due to respiratory viral infections, exposures to fungal aeroallergens, endotoxin that is contained in dust that also contains allergens, and many other factors. Comorbidities, including obesity, may also increase non‐type 2 inflammation. Both type 2 and non‐type 2 inflammation are suppressed by T regulatory cells (Tregs). Tregs express the transcription factor Foxp3 and suppress airway inflammation by limiting effector function of Th1, Th2, Th17, and other cells [25, 26, 27]. In this review and summarized in Figure 2, we will discuss how sex hormones and sex chromosomes modify pathways that are imperative for driving type 2 and non‐type 2 inflammation.

## 2 Sex Differences in Childhood Asthma

Asthma and acute asthma exacerbations are a significant cause of morbidity and mortality among children, affecting over 6.5 million children in the United States, and its incidence is increasing globally [28, 29]. Additionally, approximately 80% of asthma onset occurs during childhood, providing a period to focus on to reduce the number of people with asthma [30, 31]. Many risk factors are linked to the development of childhood asthma, including pre‐ and perinatal exposures [32, 33, 34, 35, 36, 37] and early childhood respiratory infections [38, 39], particularly infection with respiratory syncytial virus (RSV) [40, 41]. Maternal asthma is also associated with an increased risk of childhood asthma, more so than paternal asthma [37, 42, 43, 44], suggesting a genetic predisposition as well as in utero mechanisms driving the onset of asthma. However, the mechanisms by which in utero exposure increases childhood asthma and sex differences to in utero exposures and the impact on risk for developing asthma remain unclear.

Asthma prevalence and severity being higher in boys compared to girls prior to puberty has several explanations. Smaller airway size to lung volume, dysanapsis, is speculated to cause increased asthma in boys compared to girls [2]. Additionally, boys have increased allergic sensitization and higher IgE production compared to girls as well as increased inflammatory responses to viral infections [45]. Many immune genes, including *TLR7*, *TLR8*, *FOXP3*, *CXCR3*, *CD40LG*, *IL2RA*, are located on the X chromosome. In females, *XIST* is a gene that is responsible for inactivating genes on one of the X chromosomes. However, incomplete X‐linked inactivation by XIST occurs approximately 15% of the time in some cell types, including immune cells [46, 47]. This leads to increased expression of X‐linked genes in females compared to males, leading to sex differences due to sex chromosomal differences in immune or other physiological responses; providing another explanation for sex differences in asthma risk and prevalence at a time when sex hormone levels are low.

As mentioned earlier, TSLP is an alarmin cytokine that is important for increasing type 2 inflammation in response to allergen exposure and non‐type 2 inflammation in response to respiratory viral infections. Recently, female mice infected with respiratory syncytial virus (RSV) in early life had increased type I IFN anti‐viral response compared to male mice [48]. RSV‐infected male mice further retained increased lung TSLP production after the virus cleared, and the male mice had more pronounced inflammatory responses to subsequent allergen exposure in adulthood [48]. While genes encoding for TSLP or the TSLP receptor are not found on the X chromosome, it is possible that epigenetic regulators of TSLP or the delayed clearance of the respiratory viruses in male versus female pups are responsible for the sex differences in the elevation of TSLP and subsequent effects with allergen exposure as adults. Additional studies need to be conducted to determine the mechanisms by which incomplete X‐linked inactivation and/or sex chromosomal differences in immune responses to environmental exposures and pathogens may differentially shape asthma rates in boys and girls during childhood.

In utero allergen exposure results in increased asthma risk in offspring, but currently sex differences in immune responses based on in utero exposures have not been determined. In mouse models, pups born to house dust mite (HDM)‐exposed moms had increased AHR, airway inflammation, and immunoglobulin and Th2‐associated cytokine production following repeat HDM exposure [49]. However, when dams were challenged with fluorescently labeled HDM, no HDM or HDM‐specific proteins, DerP1 or DerP2, were detected in the placental and amniotic fluid. These data suggest there is minimal to no HDM transfer to fetuses, and that in utero HDM‐induced changes to immune responses in offspring are not from direct contact with allergen [49]. Recent data in mouse models with dams exposed to diesel exhaust pollution during pregnancy showed no sex differences in elevated NK‐mediated immune responses in male and female pups [50]. These data currently suggest no sex differences to in utero aeroallergen or pollutant exposures. However, more extensive studies are needed.

In human longitudinal birth cohorts, sex differences are observed in asthma prevalence. Recently, it was reported that chronic stress during pregnancy increased the risk for asthma more in girls compared to boys [51]. Other studies tracking the timing of first RSV viral infection and the risk of asthma found no differences in boys and girls [52], yet the impact of RSV infection on immune cell development and function or on airway epithelial cell differentiation has yet to be determined. Large cohorts with longitudinal sampling of immune cells and/or nasal airway epithelial cell differentiation are needed to determine sex differences in immune cell development in response to respiratory environmental exposures.

## 3 Sex Differences in Asthma in Adulthood

Prior to puberty, asthma prevalence and severity are higher among males compared to females at a 2:1 ratio, and this relationship switches after puberty. Rapid increases in androgens in pubescent males are associated with a decline in asthma prevalence and symptoms and an increase in lung function in the Severe Asthma Research Program cohort [53]. Additionally, fluctuations in the ovarian hormone levels are linked to increased asthma incidence in adult females [54]. These cohort analyses suggest that sex hormones are also important in driving asthma pathogenesis. A recent meta‐analysis study examined the gene expression differences in women and men with asthma using a large gene expression database (*n* = 3639 patients with asthma, 56% female) and a validation cohort of *n* = 132 patients with asthma, 78% female. In this study, 61 genes were differentially expressed in circulating immune cells in women and men with asthma that correlated with clinical features in asthma [55]. *IL7R*, a component of the TSLP receptor, as well as *ICOS* and *IL2RA*, genes important in type 2 inflammatory responses, were increased in females with asthma compared to males with asthma. These results using large asthma gene datasets show a sex difference in gene expression associated with type 2 inflammation in patients with asthma.

In women, asthma control and asthma symptoms can fluctuate during the menstrual cycle, pregnancy, and after menopause. Perimenstrual worsening of asthma affects up to 40% of women with asthma and results in increased asthma morbidity, including ED visits, hospitalization [56, 57, 58]. The mechanisms driving perimenstrual asthma are not well characterized. During the perimenstrual phase of asthma, estrogen and progesterone levels are reduced, and so it remains unclear if the low levels or the fluctuations of estrogen and progesterone alter airway inflammation and AHR.

In pregnancy, asthma symptoms and control worsen in approximately 40% of the cases [59], particularly in pregnant women with more severe asthma. Asthma symptoms during pregnancy may be altered by increased estrogen and progesterone levels, a more pronounced type 2 inflammatory response, and/or reduced lung capacity due to expanding uterus [60, 61]. Interestingly, the sex of the fetus may also impact asthma symptoms during pregnancy, with women carrying female fetuses having increased asthma symptoms compared to women carrying male fetuses [62, 63, 64].

Menopause, defined as no menstruation for 12 consecutive months, also modifies asthma prevalence and asthma symptoms. During menopause, estrogen and progesterone levels are decreased, with increases in follicle‐stimulating hormone (FSH) and luteinizing hormone (LH). Conflicting data exist on the impact of menopause on asthma prevalence and symptoms, with some studies showing increased asthma prevalence after menopause and others showing no difference in asthma prevalence pre‐ and post‐menopause [65, 66, 67]. These differences in study outcomes may be due to the complex nature of defining and tracking menopause in large cohort studies. Hormone replacement therapy is also prescribed for women during menopause, and a meta‐analysis showed no association of hormone replacement therapy use on asthma symptoms or asthma control [67]. Additional studies in pre‐clinical mouse models are needed to understand the mechanisms of how fluctuations and changes in estrogen and progesterone affect airway inflammation and AHR. However, this is hard to replicate in mice, as mice do not have a menstrual cycle or undergo menopause similar to what is observed in humans. Chemical atresia of the follicles in the ovaries using 4‐vinylcyclohexene diepoxide (VCD) has been used in mice to mimic menopause, since VCD results in low estrogen and progesterone levels with increased FSH and LH levels [68]. Allergen exposure after VCD administration showed increased neutrophil infiltration and AHR compared to mice administered vehicle control [69, 70]. These data start to untangle how menopause impacts airway inflammation, AHR, and asthma control. Yet more studies need to be conducted using large human longitudinal data and mouse models with VCD treatment.

## 4 Sex Hormones Modify Airway Inflammation in Asthma

While much is known about sex differences in asthma incidence, prevalence, and asthma exacerbations, little is known about how sex chromosomes and XIST impact airway inflammation and AHR. As discussed below, more information is available on how sex hormones impact asthma pathogenesis. Estrogen signaling through the estrogen receptor (ER)‐α increased type 2 and Th17‐mediated airway inflammation in asthma [15, 71, 72]. ER‐α signaling also increased IL‐17A protein expression and mitochondrial metabolism in Th17 cells and pathogenic Th17 cell differentiation [72]. Selective activation of ER‐α with an agonist, propyl pyrazole triol (PPT), also enhanced mitochondrial function and systemic metabolism in high fat diet (HFD), ovariectomized (OVX) female mice compared to HFD sham operated and HFD OVX mice given a vehicle [73]. Yet ER‐α effects did not directly impact Th2 cells since Esr1^fl/fl^ X Cd4^Cre+^ mice that lack ER‐α signaling specifically in T cells had similar lung Th2 cells and eosinophil infiltration after HDM treatment [74]. Additional studies showed that ER‐α signaling increased type 2 macrophages (M2 macrophages) both in vitro using bone marrow derived macrophages and in vivo in ovariectomized female mice with continuous release estradiol pellet undergoing OVA‐induced allergic airway inflammation [75]. However, in female mice ovariectomized after adulthood that were subsequently administered continuous release estradiol pellets dosed to physiological levels, no changes in eosinophil or neutrophil recruitment to the airways were detected following HDM challenge in the ovariectomized mice administered vehicle or estradiol pellets [76]. These data indicate that estrogen and ER‐α signaling have long‐lasting epigenetic effects on M2 differentiation and airway inflammation and that the timing of ovariectomy and the concentration of estradiol rescue pellets is important to consider when designing mouse models directed at testing the impact of estrogen signaling on airway inflammation.

Androgens signaling through AR decreased type 2 and Th17‐mediated airway inflammation while increasing T regulatory (Treg) cell suppressive function in asthma [77, 78, 79]. We have published that androgens signaling through the androgen receptor (AR) were important in downregulating allergen‐induced airway inflammation in mouse models as well as increasing lung function, measured with FEV1, in patients with asthma administered the androgen DHEA [74, 77, 80]. In addition, patients with androgen insensitivity syndrome had increased asthma prevalence compared to controls [81]. Increased AR gene expression in bronchial brushings was also associated with better lung function and fewer asthma symptoms and that mutations in β‐hydroxysteroid dehydrogenase‐1 (3β‐HSD1) that restricted conversion of DHEA into downstream androgens were associated with glucocorticoid resistance asthma in the SARP cohort [82, 83]. AR signaling also decreased mouse and human airway epithelial cell IL‐33 production and limited allergen‐induced eosinophil and neutrophil infiltration in the airways of mice [74, 77, 78, 79]. Further, AR signaling decreased CD4+ Th2 cells, CD4+ Th17 cells, and ILC2 cell potentiation or differentiation as well as reduced IL‐5, IL‐13, and IL‐17A cytokine production while increasing T regulatory (Treg) suppressive function and stability [74, 77]. In contrast, AR signaling increased differentiation of type 2 alveolar macrophages and reduction of AR signaling from monocytes and macrophages in *Ar* ^floxed^ X *Lysm* ^Cre+^ mice reduced eosinophil infiltration, IL‐5 production, and gene expression of *CCl11* and *CCl22* (eotaxin 1 and 2, respectively) in OVA sensitized and challenged mice [84]. Collectively, these findings show that AR signaling attenuated allergic airway inflammation by directly attenuating Th17 cells, ILC2, and IL‐33 release from AECs, but AR signaling also increased type 2 macrophages.

T cell metabolism is essential for determining the differentiation and effector for CD4 T cell subsets, Th1, Th2, Th17, or T regs. Our prior work has shown that AR signaling modifies CD4 T cell metabolism in both humans and mice [74, 85]. Upon activation, all CD4 T cell subsets increase reliance on glycolysis, but Th17 and Th2 cells also rely on glutaminolysis and one carbon metabolism for maximal effector function [74, 85, 86, 87]. Our prior work showed that circulating CD4+ T cells from patients with asthma are more metabolically active compared to healthy controls [85], and that AR signaling decreased metabolic function, including glycolysis, mitochondrial respiration, and glutamine metabolism, in Th2 and Th17 cells from women with moderate to severe asthma [74]. In mouse Th17 cells, AR signaling decreased the expression of the glutamine transporter genes, *Slc1a5* and *Slc38a1*, to limit glutamine uptake and glutaminolysis in CD4+ Th17 cells, as well as decrease allergen‐induced airway inflammation and AHR [74]. Similar experiments were conducted in Esr1fl/fl X Cd4Cre+ female mice, which lack ER‐α signaling in T cells, and ER‐α signaling had no direct effect on glutaminolysis in Th17 cells or in modifying allergen‐induced airway inflammation [74]. These data show elevated CD4 T cell metabolism in asthma and that Th17 cells from women have an increased reliance on glutaminolysis compared to Th17 cells from men—providing mechanistic insight on how sex hormones are altering CD4 T cell effector function in asthma pathogenesis.

## 5 Obesity and Asthma

Obesity is associated with increased asthma prevalence and incidence, and obese women have increased asthma prevalence compared to non‐obese women, non‐obese men, and obese men [88]. Additionally, obese women are more likely to have increased asthma symptoms and decreased lung function when taking hormone replacement therapy during menopause [89]. Studies have also shown that lifestyle weight loss and decreased body mass index cause significant improvement in asthmatic symptoms, increased lung function, and decreased numbers of asthma exacerbations [89]. Additionally, several studies have shown that obese male mice had greater airway inflammation compared to non‐obese male mice [90, 91]. Obesity studies in female mice have been limited due to lack of weight gain in female mice on high fat diets (HFD) compared to male mice on HFDs. Recent studies by our group showed that HFD‐fed female mice had increased neutrophilic inflammation that was dependent on ovarian hormones [92]. In particular, ER‐α signaling in T cells was important for increasing neutrophil infiltration and lung Th17 cells in HFD female mice since *Esr1* ^fl/fl^ X *Cd4* ^Cre+^ female HFD mice had reduced neutrophils and lung Th17 cells after HDM exposure compared to *Esr1* ^fl/fl^ female mice [92]. These data were interesting; ER‐α signaling had no impact on allergen‐induced airway inflammation was determined in female mice on normal chow diet, as discussed above [74], only in HFD‐fed female mice.

## 6 Sex Differences in Viral Infections

Viral infections are important drivers of asthma exacerbations or asthma attacks. Sex differences in incidence, morbidity, and mortality to respiratory viral infections between men and women also occur and are partially dependent on age, virus, and presence of androgens and/or estrogens. Women ages 20–49 have increased morbidity and mortality in response to influenza infections compared to men of the same age [93]. Yet, prior to 20 and after 70+ years, men have increased morbidity and mortality to influenza [93]. The increased incidence, morbidity, and mortality in men greater than 70 has been posited to be associated with a decline in androgens and AR signaling [93]. In contrast, men aged 45–70 have increased COVID 19 morbidity and mortality associated with COVID19 compared to women. Additional studies with experimental infection with RSV A Memphis 37 strain showed that only 50% of men were qPCR+ for RSV compared to women that had 60% positivity [94, 95, 96]. Yet, how respiratory viral infections differentially affect men and women with asthma to lead to increased asthma symptoms and/or asthma exacerbations is unknown and more evaluation is needed.

## 7 Conclusion

There is a sex difference in asthma prevalence and asthma‐related morbidity throughout life. Using large cohort (human) studies, human cells, and mouse models of asthma, the impact of sex hormone signaling on asthma pathogenesis has been partially elucidated. Androgens and AR signaling decrease airway inflammation by reducing alarmin production, resulting in less type 2 inflammation, as well as directly reducing Th17 cell differentiation and neutrophil‐induced airway inflammation. Estrogen signaling through ER‐α increases IL‐33 production from airway epithelial cells to increase type 2 inflammation and increases Th17 cell differentiation. In high‐fat diet females, ER‐α signaling in T cells is also important in increasing allergen‐induced neutrophil infiltration. Additional studies examining the impacts of sex chromosomes on modifying immune responses in airway inflammation need to be conducted as well as more extensive studies on sex differences in immune cell development and airway inflammation during childhood. Collectively, understanding that sex differences in asthma risk and control exist throughout life is important for clinicians to recognize in personalizing therapies and treatment options for males and females with asthma.

## Conflicts of Interest

The authors declare no conflicts of interest.

## Data Availability

The authors have nothing to report.
